# Management of the “building-base system” action, using a life cycle model

**DOI:** 10.1038/s41598-021-98367-0

**Published:** 2021-09-22

**Authors:** Justyna Sobczak-Piąstka, Oksana Kichaeva, Pavlo Firsov, Sergey Zolotov, Yuriy Famulyak

**Affiliations:** 1 Faculty of Civil and Environmental Engineering and Architecture, Bydgoszcz University of Science and Technology, Bydgoszcz, Poland; 2 Department of Soil Mechanics, Foundations and Engineering Geology, O. M. Beketov National University of Urban Economy in Kharkiv, Kharkiv, Ukraine; 3 Department of Building Structures, O. M. Beketov National University of Urban Economy in Kharkiv, Kharkiv, Ukraine; 4 Faculty of Civil Engineering and Architecture, Lviv National Agrarian University, Zhovkva district, Dubliany, Lviv Region Ukraine

**Keywords:** Civil engineering, Environmental impact

## Abstract

This research aims to highlight the problem of creating various models that reflect the life cycle of the “building-base” system, and link the reliability of this system with its technical condition. Based on the proposed model of the life cycle “reconstructed building-base”, it is possible to regulate the condition of the building towards its restoration, changing the category of technical condition to higher and obtain new, adequate to the current state, reliability indicators. During the operation of this model, it is possible to control the system management on the basis of the obtained quantitative values of reliability. The result of the reliability calculation is the determination of such a technical solution of strengthening the “building-base” system, which will provide a given level of reliability. The research provides an example of the calculation of an operable monumental building before and after its restoration.

## Introduction

In Ukraine constantly increase the number of buildings that are in critical condition and require major repairs with structures strengthening. Analysis of accidents causes of brick structures in the “building-base system” shows the necessity to control and manage the reliability and serviceability at all stages of the life cycle. Today there is an obvious necessity for new science-intensive models for assessing the reliability of building structures both at the level of design, operation, and in normative regulation. There is also a lack of research on the development of probabilistic models of the system “building-base system”, which would determine the resource of the system at any time of the life cycle, so the problem of developing such models is an urgent task for today.

The task of determining the reliability of the “building-base system”, especially which has been in operation for a long time and the components of which can be reconstructed, can be formulated as follows:

- implementation of an adequate calculation model of the “building-base system” in terms of reconstruction. If sufficient tools for such modeling have already been developed for new construction, then for the reconstructed objects there are many more features that should be taken into account in a single calculation scheme. These include, for example, the existence of damaged structural elements of the building before reconstruction (cracks, reduction of the elements cross-sections), deformations of the foundation, which exist at the time of reconstruction, changes in operating conditions of the building, changes in engineering and geological conditions;
- detection and description of changes in working conditions—loads, properties of building materials and soils, etc. Quantitative description of most of these factors in the complexity of their interaction should be carried out taking into account the variability of soil properties of bases and materials, as well as loads and influences;
- selection of the model for assessing the reliability of the “building-base system” during operation;
- application of the selected model (method) to assess the technical condition of the “building-base system” and determination of the system resource.

Numerous publications of both Ukrainian and foreign scientists are devoted to the management of the structures action. It is necessary to note among them the monographs of Anatolii Perelmuter^1,2^, scientific works of Valerii Shmukler^3,4^, Sergiy Pichugin^5^, Anatolii Roytman^6^, Andrey Dobromislov^7^, Aleksandr Lychev^8^, Albert Lantoukh-Lyashchenko^9^, Oksana Kichaeva^10^ and others.

Throughout the life cycle of the “building-base system”, the technical condition of the elements of this system changes for the worse under the influence of many factors. These factors negatively affect the physical and mechanical characteristics of the components of the system, which leads to various types of damage and deformation, resulting in a decrease in the reliability of the components of the system, its safety and durability.

One of the problems is the connection of the technical condition of the structure with the reliability, which is expressed by a numerical parameter, which serves as a quantitative integrated assessment of the technical condition of the system. If this problem is solved and an adequate life cycle model is selected (or developed), the question arises of restoring the technical condition of the system to an acceptable one, using a probabilistic approach.

The main purpose of the research is to apply proposed model of the life cycle “reconstructed building-base”, which was tested on a real object in Kharkiv, Ukraine. During the operation of this model, it is possible to control the system management on the basis of the obtained quantitative values of reliability.

The proposed model can be used for any buildings in all regions, since its components are determined exceptionally by real parameters that determine the condition of “building-base system”: service life, geotechnical conditions, conditions of technical operation of the system, recovery factor.

## Basic material

Within the framework of this research, the reliability and technical condition of the “building-base system” for the historic building in Kharkiv was assessed using the author's life cycle model and measures to restore the reliability of the considered systems were proposed.

**Figure 1:**
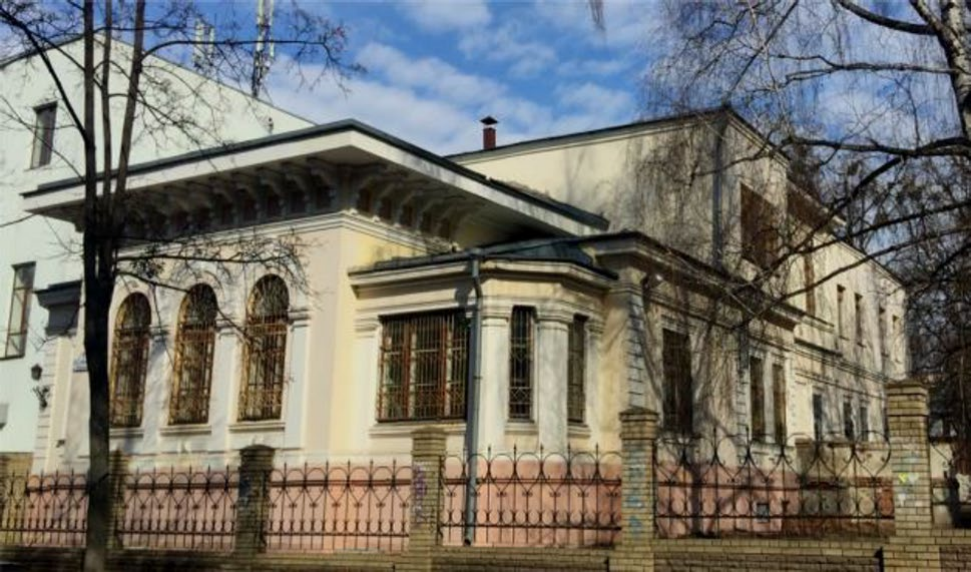
Main facade of the building, modern view.

Monument of architecture and urban planning of local significance on the Skrypnika str., built according to the project of academician O.M. Beketov in 1897 as a residential building. In the 1960-s, this building was reconstructed according to extension and partial addition of the second floor. The building has a rectangular form, consisting of a one-storey tier with dimensions in plan 10.0 × 18.0 m, and a two-storey tier with dimensions in plan 25.8 × 12.9 m, also there is a basement and an attic. Structural solution of the building—frameless building, with load-bearing brick longitudinal and transverse walls, combined overlaps (vaulted basement and beam wooden—interfloor); in the two-storey tier—two-span with load-bearing longitudinal walls (Fig. 1).

The walls of the building are made of red clay bricks with a chain seams bandage. Brickwork of wedge-shaped bulkheads, vaults, internal walls of the basement—on a cement-sand mortar, walls—on a lime and in locale places with a cement-sand mortar; thickness of external bearing walls is 850 mm. The brand of a brick, after carrying out destructive tests, is determined as M50. The foundations of the building are tape, brick, which is a continuation of the walls of the building. Depth of foundations from the surface ≈ 1.5–1.6 m, depth from the basement floor—varies from 0.25 to 0.7 m.

In geomorphological terms, the research area belongs to the interfluve of the Lopan and Kharkiv rivers. The relief of the site is flat, with a general slope in the south-western direction, planned by bulk soils with capacity up to 2.7 m thick. Quaternary bulk loams, loams and sands of small and medium density, covered with bulk soils with an admixture of plant residues, take part in the geological structure of the building site.

Under-basement floor in several rooms—is brick vaults 120 mm thick, bearing on metal rails with a step of 1 m, bearing on brick walls. Floors in local part of the basement—are reinforced concrete slabs on a brick on metal beams. The interfloor overlap—is wooden. The roof system of different parts of the building is a system of layered (2-storey tier) and hanging wooden rafter structures (1-storey tier).

**Figure 2:**
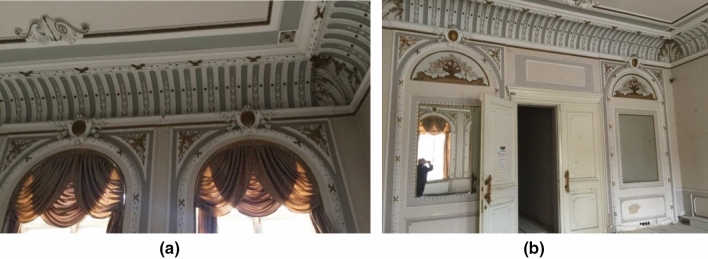
Interior decoration of the hall of the building: (**a**) windows, cornices; (**b**) mirrors, picturesque inserts above them, various doors.

The wall of the main facade is elegantly decorated—the walls and partitions are decorated with rusts, panels, locks over the windows, cornices, stucco details. The cornice of the one-storey tier is made far beyond the outer edge of the facade, the cornice is supported by high brackets, between which there are square panels with stucco in the form of stylized lilies. Facades windows are framed by an overhead arcade, which consists of profiled archivolts and keystones. Interior walls of the building are decorated with stucco, cartouches, mirrors, picturesque inserts (Fig. 2).

The inspection revealed numerous defects and damages of the building: cracks of force and draft origin in the load-bearing walls and vaults, local destruction of brickwork, corrosion of reinforcement and destruction of the protective layer of the basement, corrosion of metal elements of reinforced concrete and vaulted floors.

**Figure 3:**
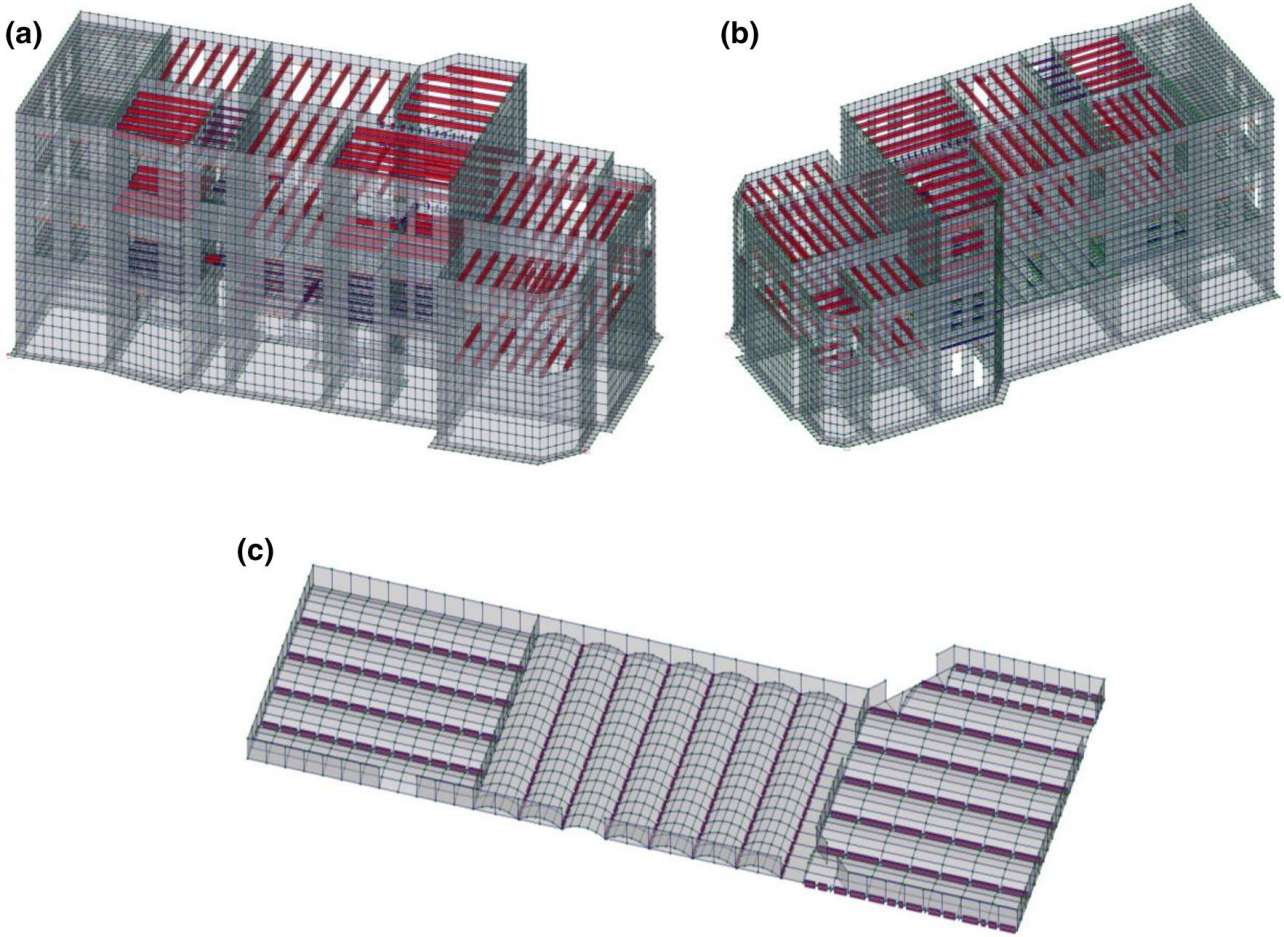
Design scheme of the building: (**a**) view from the main facade; (**b**) view from the side of the courtyard facade; (**c**) brick vaults.

To establish the real stress-deformed condition of the building, a force calculation was performed using the Structure CAD v. 21.1 software package (Fig. 3).

The calculation scheme includes finite elements of the following types:

- for walls, foundations and floor slabs—shell elements with 6 degrees of freedom in each joint;
- for tape foundations—elements of the type of slab on an elastic basis with three degrees of freedom in each joint;
- for reinforced concrete bulkheads, metal and wooden beams of the overlap and roof—rod finite elements with 6 degrees of freedom in each joint.

To solve the problem of determining the stress-deformed condition of the building in its interaction with the soil base, a two-parameter model of Pasternak P.L. was used, which takes into account the soil work on compression and the distribution capacity of the soil. In this regard, in the elements that are in direct contact with the base, these coefficients of the elastic base were introduced. The coefficients of the elastic base were taken depending on the specific engineering and geological conditions of the site of the surveyed building. The rigid characteristics of the elements were set in accordance with the results of the survey and regulations. For the elements, modeling a brick masonry, the deformation modulus was defined according to results of brick tests and instructions of State Building Normatives of Ukraine and State Standards of Ukraine (SBN “Stone and reinforced stone constructions”^11^ and SSTU “Calculation and design of stone and reinforced stone constructions of buildings and structures”^12^).

**Figure 4:**
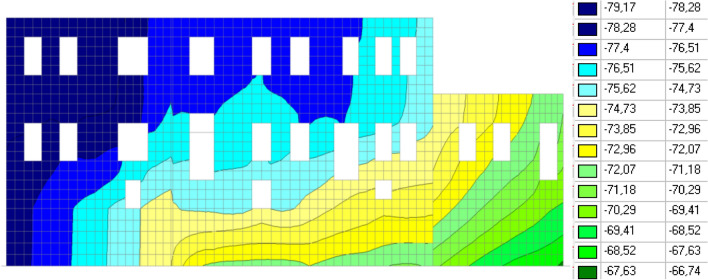
Vertical displacements of the building, mm.

**Figure 5:**
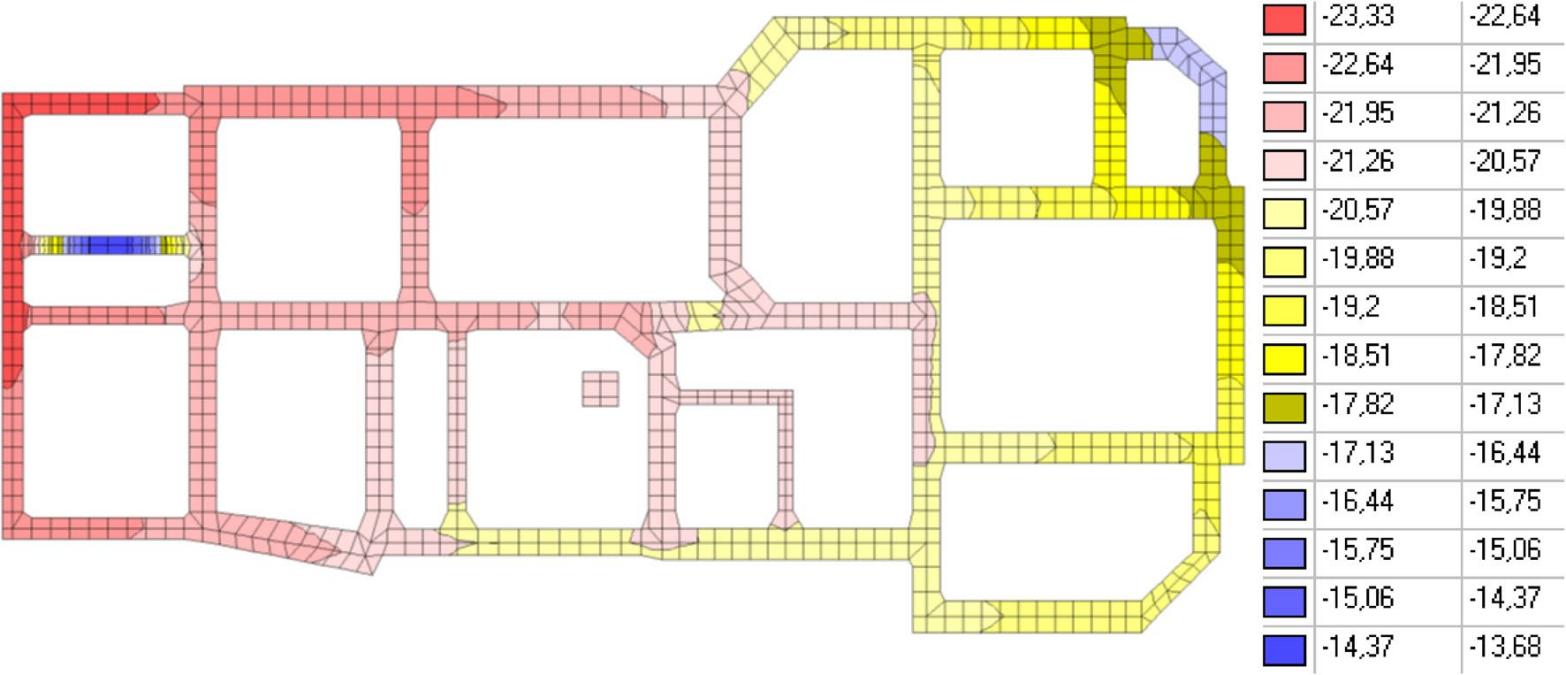
Soil resistance, t/m^2^.

**Figure 6:**
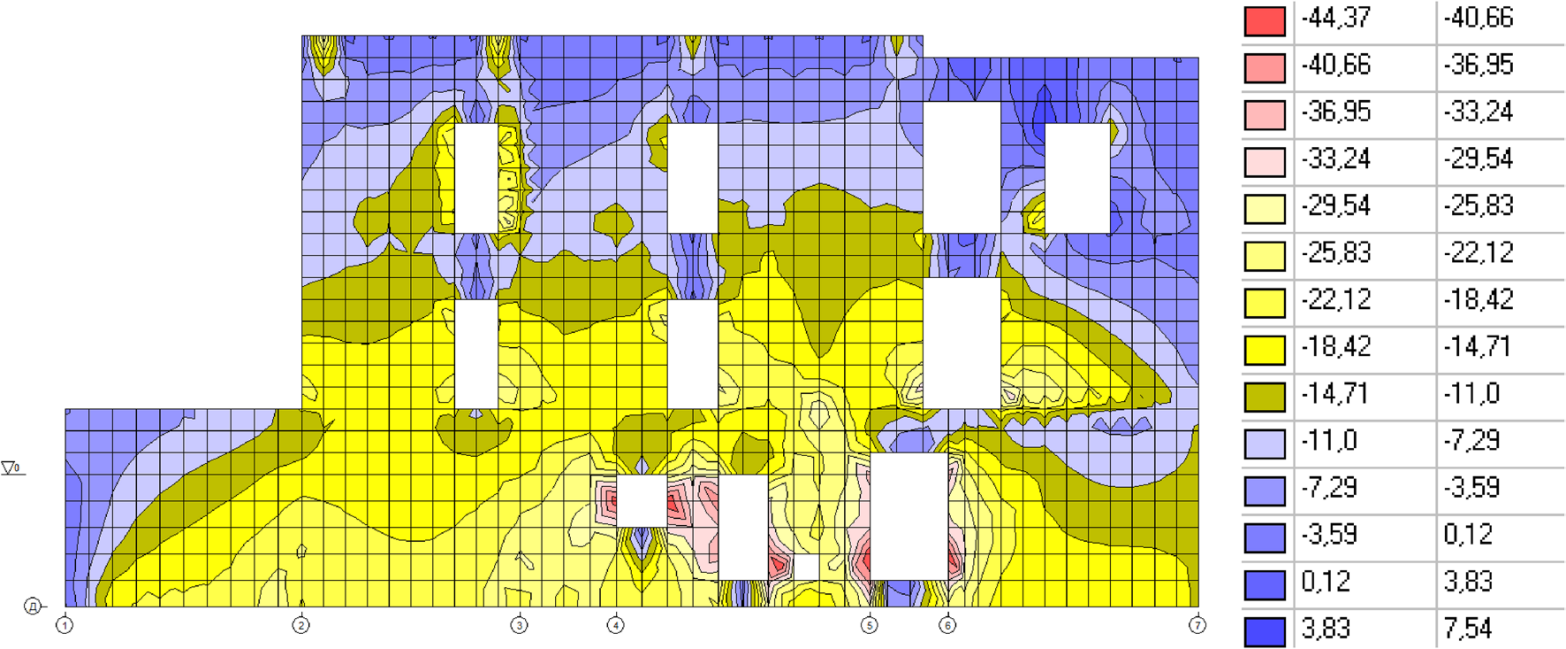
Vertical normal stresses in a brick wall along the “E” axis, t/m^2^.

The results of the calculations are presented in Figs. 4, 5, and 6.

### Ethical statement

Authors state that the research was conducted according to ethical standards.

## Research results

After inspection and carried out calculations, the technical condition of the “building-base system” was determined, which was formulated as unsuitable for further operation (category of technical condition 3) due to unsatisfactory condition of brick load-bearing walls and brick vaults. Now, it is necessary to determine the reliability *P*(*t*) and the value of reliability index β (safety characteristics) of the system in accordance with proposed with the proposed life cycle model. The main provisions of this model are set out in paper^10^. Theoretically, the model is based on the following hypotheses:

1. the criterion of the system technical condition is a numerical parameter of reliability, which serves as a quantitative integrated assessment of the system technical condition;
2. the reliability of the base during the life cycle of the operation is assumed to be constant, determined at the design stage;
3. the process of system degradation during the life cycle of operation is represented by four discrete states with a uninterrupted time;
4. the process of system degradation during the life cycle is described by a nonlinear algebraic function.

Thus, as the only parameter of the model is accepted the value of the initial system reliability *P*_*d*_, obtained at the design stage. The model is deterministic, time *t* is uninterrupted during the operation life cycle. The life cycle model is reduced to a discrete form with the division of the life cycle into four discrete operating states in accordance with the regulatory documents. The model ignores the random nature of the life cycle time, i.e. with the given initial data the predicted operation time is the only fixed value. The life cycle model is presented in formula:

1 $$P(t)={P}_{d}\times f(t)$$

where: *P*_*d*_—is the calculated reliability of the “building-base system” at the design stage (the multiplication of two reliability parameters: building and base); *f(t)*—degradation indicator-function; *t*—time, years.

The degradation function is presented in the following form:

2 $$f(t)=(1-s\times {K}_{1}\times {K}_{2}\times {P}_{c}\times {P}_{d}^{-1}\times {t}^{2})$$

In addition to the fact that the model has a interconnection between the building and the base, which is determined at the earliest stage (design stage), the operating factor *K*_*1*_ is introduced, depending on the geotechnical category of operational complexity (according to Eurocode 7^13^) and takes three values: 1.0; 1.2 or 1.4; *K*_*2*_—operation coefficient, which takes the following values depending on the conditions of maintenance and service of the system “building-base system” and can take the following values 1.0; 1.05 or 1.1; *s*—dimensional coefficient of the model; *P*_c_ = *P*_*d*_ − *P*_*lim*_—reliability, the difference between design and limit values in operation.

After importing (2) in the life cycle model, we have:

3 $$P(t)={P}_{d}\times (1-s\times {K}_{1}\times {K}_{2}\times {P}_{c}\times {P}_{d}^{-1}\times {t}^{2})$$

The dimensional coefficient of the model*s* is expressed in initial constant—the design operation time of the building *T*_*d*_ :

4 $$s=\frac{1}{{T}_{d}^{2}}$$

where: *T*_*d*_—design operation time of the building, years.

The value of the established operation time *T*_*ef*_ is taken according to SBN “System for ensuring reliability and safety of building objects. General principles of security reliability and constructive safety of buildings, structures, building constructions and bases”^14^.

If the building has exhausted its operation time, after which it has undergone major repairs and reconstruction with full restoration of the load-bearing capacity of the main structures, replacement of outworn structures with new ones and complete replacement of networks, equipment and complete restoration, in our opinion, in this case, in formula (3) it is advisable to enter another coefficient *K*_*3*_—recovery coefficient, depending on the ratio *P*_*d*_ (calculated reliability of the “building-base system” at the design stage) to *P*_*lim*_—the limit value of reliability in operation as follows:

5 $${K}_{3}=\frac{{P}_{d}}{{P}_{lim}}$$

So, the formula (3) will have next view:

6 $$P(t)={P}_{d}\times (1-s\times {K}_{1}\times {K}_{2}\times {K}_{3}\times {P}_{c}\times {P}_{d}^{-1}\times {t}^{2})$$

**Figure 7:**
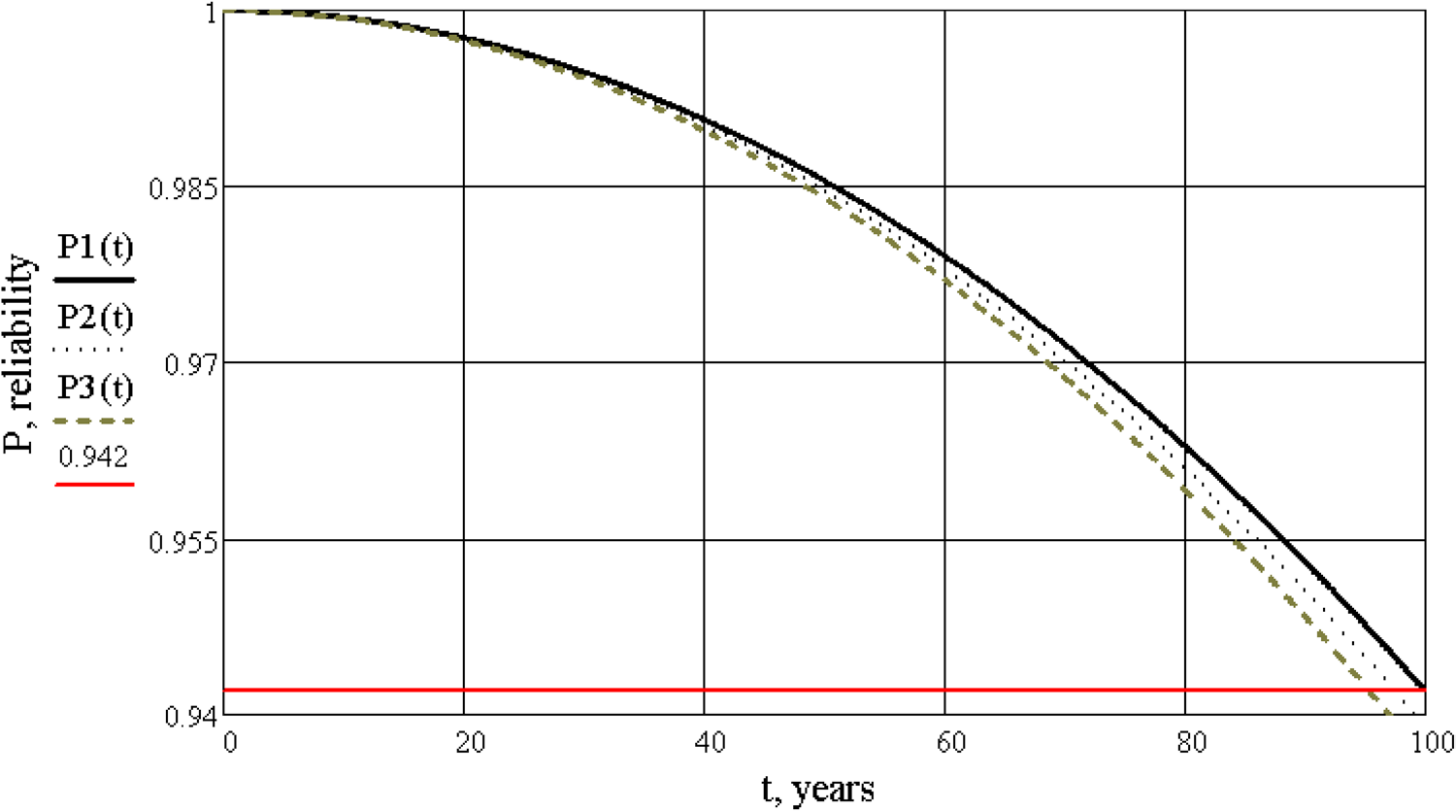
Graphs of reliability dependence of the “building-base system” on time, during a standard cycle of operation.

**Figure 8:**
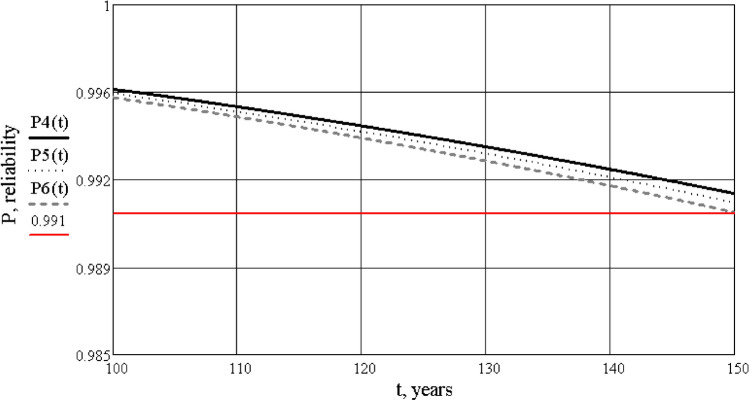
Graphs of reliability dependence of the “building-base system” on time, after reconstruction.

**Figure 9:**
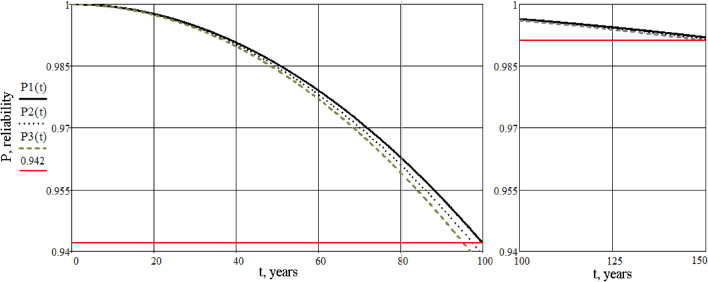
Combined graphs of reliability dependence of the “building-base system”, before and after reconstruction.

Then, it is necessary to calculate the values of reliability and plot the dependence of reliability graphs, depending on time during the regulatory cycle of operation, and after full recovery with partial restoration of reliability (Figs. 7, 8, 9). The calculation is performed using the Mathcad software according to a pre-compiled algorithm. Reliability is determined by the reliability index β (or safety characteristic), which is defined as β = − ${\Phi }^{-1}({p}_{f})$, where ${\Phi }^{-1}$ is the inversion of the standard normal distribution (according to ISO 2394:2015^15^).

## Conclusions

Analysis of the obtained information leads to the following conclusions:

- the main result of the research is the development of a life cycle model “reconstructed building-base”, the parameters of which are the technical condition and its reliability. During application of this model it is possible to control the condition of the system based on the obtained quantitative values of reliability;
- the calculation of the “building-base system”, using the finite element method (SCAD software), which allowed to determine the stress-deformed condition and the category of technical condition of the building, not only by visual and instrumental inspection, but also to confirm the calculations as required by Ukrainian national norms, was completed;
- the reliability of the “building-base system” for the reconstructed building in the Mathcad software was calculated. It is shown that as its service life is exhausted and damage accumulates, the reliability of the building is equal to $P\left(t\right)=0.94$, which corresponds to an emergency or unusable condition. Restoration of the building raises the level of reliability to an acceptable value $P\left(t\right)=0.991$, which corresponds to a satisfactory condition of the system;
- according to the results of the calculation by finite element method and the calculation of reliability, a coincidence was evaluated in determining the category of technical condition of the system, which confirms the acceptability of the proposed model for application in this field;
- the result of the calculation of the reliability definition is the choice of the correct technical solution to strengthen the “building-base system”, which will provide a given level of reliability. The research provides an example of the calculation of the existing architectural monument before and after its restoration;
- further development of such models makes it possible to manage the reliability of the system—to anticipate and eliminate potential negative consequences during the operation of the “building-base system” for buildings of different types and purposes.

## References

[1] Perelmuter AV (2011). Management of Bearing Constructions Action.

[2] Perelmuter AV (2007). Selected Problems of the Reliability of Building Structures.

[3] Shmukler VS, Gorodetskii AS (2002). Formation of Design Schemes in Conditions of Structures Stress-Deformed Condition Regulation.

[4] Babayev V (2019). Rational Design of Structural Building Systems.

[5] Pichugin S, Klochko L (2019). Accidents features in construction. Acad. J..

[6] Roytman AG (1990). Residential Buildings Accidents Prevention.

[7] Dobromislov AN (2004). Assessment of the Reliability of Buildings by External Signs.

[8] Lychev AV (2008). Reliability of Building Structures.

[9] Lantoukh-Lyashchenko A (2016). The problem of bridge security normative management. Bridges Tunnels.

[10] Kichaeva, O. Operation life-cycle model of the “building-base” system. in Proceedings of CEE 2019 “Advances in Resource-saving Technologies and Materials in Civil and Environmental Engineering, 153–160. 10.1007/978-3-030-27011-7_19 (2019).

[11] SBN V.2.6-162:2010. *Stone and Reinforced Stone Constructions*. (Ministry of Regional Development and Building of Ukraine, 2011).

[12] SSTU B V.2.6-207:2015. *Calculation and Design of Stone and Reinforced Stone Constructions of Buildings and Structures*. (Ministry of Regional Development of Ukraine, 2016).

[13] SSTU-N B EN 1997-1:2010. Eurocode 7. Geotechnical design. Part 1. General rules (EN 1997-1:2004, IDT). (Ministry of Regional Building, Development and Municipal economy of Ukraine, 2014).

[14] SBN V.1.2-14-2018. *General Principles of Ensuring the Reliability and Structural Safety of Buildings and Structures*. (Ministry of Regional Development of Ukraine, 2018).

[15] ISO 2394:2015. *General Principles on Reability for Structures*. (International Organization for Standardization, 2015).

